# MAECI: A pipeline for generating consensus sequence with nanopore sequencing long-read assembly and error correction

**DOI:** 10.1371/journal.pone.0267066

**Published:** 2022-05-20

**Authors:** Jidong Lang

**Affiliations:** Department of Bioinformatics, Qitan Technology (Beijing) Co., Ltd, Beijing, China; Chinese Academy of Sciences, CHINA

## Abstract

Nanopore sequencing produces long reads and offers unique advantages over next-generation sequencing, especially for the assembly of draft bacterial genomes with improved completeness. However, assembly errors can occur due to data characteristics and assembly algorithms. To address these issues, we developed MAECI, a pipeline for generating consensus sequences from multiple assemblies of the same nanopore sequencing data and error correction. Systematic evaluation showed that MAECI is an efficient and effective pipeline to improve the accuracy and completeness of bacterial genome assemblies. The available codes and implementation are at https://github.com/langjidong/MAECI.

## 1. Introduction

Long reads from nanopore sequencing platforms such as Oxford Nanopore Technologies (ONT) are widely used in the study of bacterial genomes [1]. Compared with short reads from next-generation sequencing (NGS), long reads can span larger genomic repeats and complex genomic structures, thus facilitating downstream genome assembly and analysis [2, 3]. Many software or algorithm have been developed for bacterial genome assembly, such as Canu [4], FlyE [5], and Wtdbg2 [6]. They have relative advantages and disadvantages as well as varying performance and assembly outcomes, but in terms of overall performance, FlyE and Raven [7] stands out as the best bacterial genome assembler [8–10]. Nanopore sequencing data are characterized by the presence of indels, non-random systematic errors [11] and the occurrence of assembly errors spanning hundreds of bases [8], which may lead to inaccurate or incomplete assemblies. Hybrid assembly, which uses both short and long reads from next- and third-generation sequencing platforms, is gaining popularity [12]. Alternatively, the assemblies can be corrected using nanopore sequencing data [13] and then polished with NGS data. Both approaches can mitigate some of these problems and improve the accuracy of the assemblies, but assembly errors cannot be completely avoided [14]. Therefore, the assembly, especially of bacterial genomes, is far from perfect, and there are many details to consider and substantial room for improvement.

Since genome assembly is often the beginning of bioinformatics analysis by de novo sequencing of bacterial genomes, assembly errors may have critical implications for downstream analysis. Therefore, we develop MAECI, a pipeline that enables the assembly for nanopore long-read sequencing data of bacterial genomes. It takes nanopore sequencing data as input, uses multiple assembly algorithms to generate a single consensus sequence, and then uses nanopore sequencing data to perform self-error correction. MAECI takes advantage of the fact that different assembly algorithms produce different assembly errors for the same data, and corrects them by methods of self-correction to produce a single consensus sequence with fewer assembly error and more accurate than any of the inputs.

## 2. Materials and methods

### 2.1 The principles of MAECI and implementation

**Fig 1:**
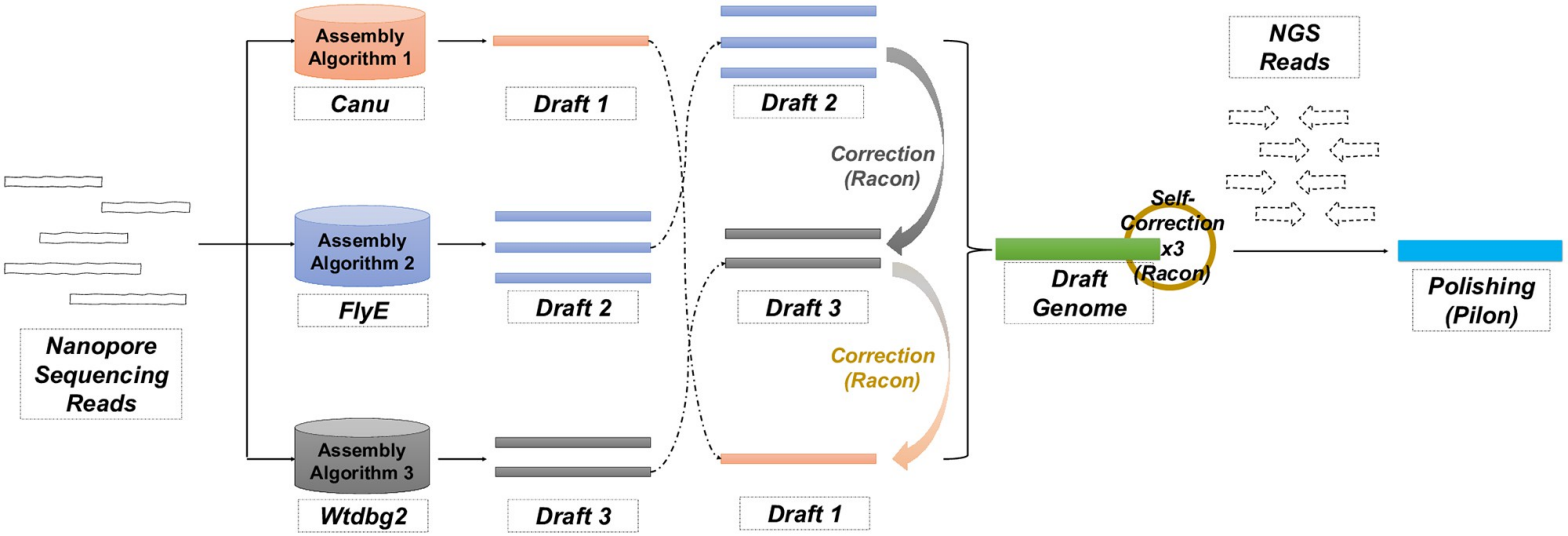
Overview of the MAECI assembly pipeline.

As shown in Fig 1, MAECI recommend that at least three assembly software and/or algorithm using long reads were used to assemble the sequencing data separately. Canu, FlyE (version: 2.8.3-b1695) and Wtdbg2 (version: 0.0 (19830203)) were selected and run with default parameters. Theoretically, the minimum number of assembly software and/or algorithm used in this step is 1, and there is no upper limit.

Then, the assembly results from various software and/or algorithm were sorted according to the number of scaffolds in descending order. Then, error correction algorithm, default is Racon (version: v1.4.20) [13], was used for “2+3” rounds of self-correction. For example, if the number of scaffolds from Canu, Wtdbg2, and FlyE was 3, 2, and 1, respectively, the sequencing data were aligned against the assembly from Canu in the first round of correction, with the assembly from Wtdbg2 used as the target genome to generate consensus sequence 1. In the second round of error correction, the sequencing data were aligned against consensus sequence 1, and the assembly from FlyE was used as the target genome to generate consensus sequence 2. Finally, the sequencing data and consensus sequence 2 were used for three rounds of error correction to obtain the final consensus sequence. Furtherly, if polishing with NGS data was required, default is Pilon (version: 1.24) [15], was used to polish the consensus sequence with default parameters.

By default, BWA (version: 0.7.17-r1188) [16] and Minimap2 (version: 2.21-r1071) [17] were installed for alignment, while Sambamba (version: 0.8.0) [18] and Samtools (version: 1.12) [19] were installed for alignment processing. NanoPlot (version: 1.38.0) [20] was used for quality control and QUAST (version: 5.0.2) [21] was used for evaluation of the sequencing data and assemblies, with default parameters.

### 2.2 Simulated-reads tests

We downloaded nine reference bacterial strain genome sequences from NCBI: *Escherichia coli* (NC_000913.3), *Pseudomonas aeruginosa* (NC_002516.2), *Campylobacter jejuni* (NC_002163.1), *Clostridium perfringens* (NC_008261.1), *Staphylococcus aureus* (NC_007795.1), *Listeria monocytogenes* (NC_003210.1), *Enterobacter cloacae* (NZ_CP009756.1), *Faecalibacterium prausnitzii* (NZ_CP030777.1) and *Enterococcus faecalis* (NZ_KZ846041.1). Then, using NanoSim-H (version: 1.1.0.4) [22], 10,000–100,000 simulated long reads were generated from each genome (S1 Table in S1 File). Using wgsim (version: 1.10) (https://github.com/lh3/wgsim), simulated NGS data with a read length of 100 bp were generated for each genome (the parameters: -d 500 -s 20 -N 5000000 -e 0.01) (S2 Table in S1 File).

### 2.3 Real-reads tests

We downloaded the real-world sequencing data published by *Lang et al*. [23] and *Wick et al*. [24]. For the *Lang et al*.’s data, it included both nanopore sequencing data and NGS sequencing data of *Alcaligenes faecalis* (*A*. *faecalis*) strain PGB1. For the *Wick et al*.’s data, the rapid sequencing and assembly results in full assemblies of three strains were selected for analysis. *trycycler_rapid_fasta*.*gz* was not found for *Serratia marcescens* 17-147-1671 dataset. For *Citrobacter koseri* MINF 9D, *Enterobacter kobei* MSB1 1B, and *Haemophilus* M1C132 1, no suitable reference genomes were available in NCBI. Thus, these three strains were excluded from analysis and comparison. As the raw ONT data were not found in the above-mentioned website, we finally downloaded the genome sequences of *Acinetobacter baumannii* strain K09-14 (NZ_CP043953.1), *Klebsiella oxytoca* strain FDAARGOS_335 (NZ_CP027426.1), and *Klebsiella variicola* strain FH-1 (NZ_CP054254.1) and used them as reference genomes. ONT data simulation was performed using NanoSim-H software. For comparison, we also performed three rounds of correction using Racon on the rapid assembly data of Trycycler [24]. QUAST was used for comparative analysis of the assembly results.

## 3. Results

### 3.1 Comparison of assembly results from simulated data

**Fig 2:**
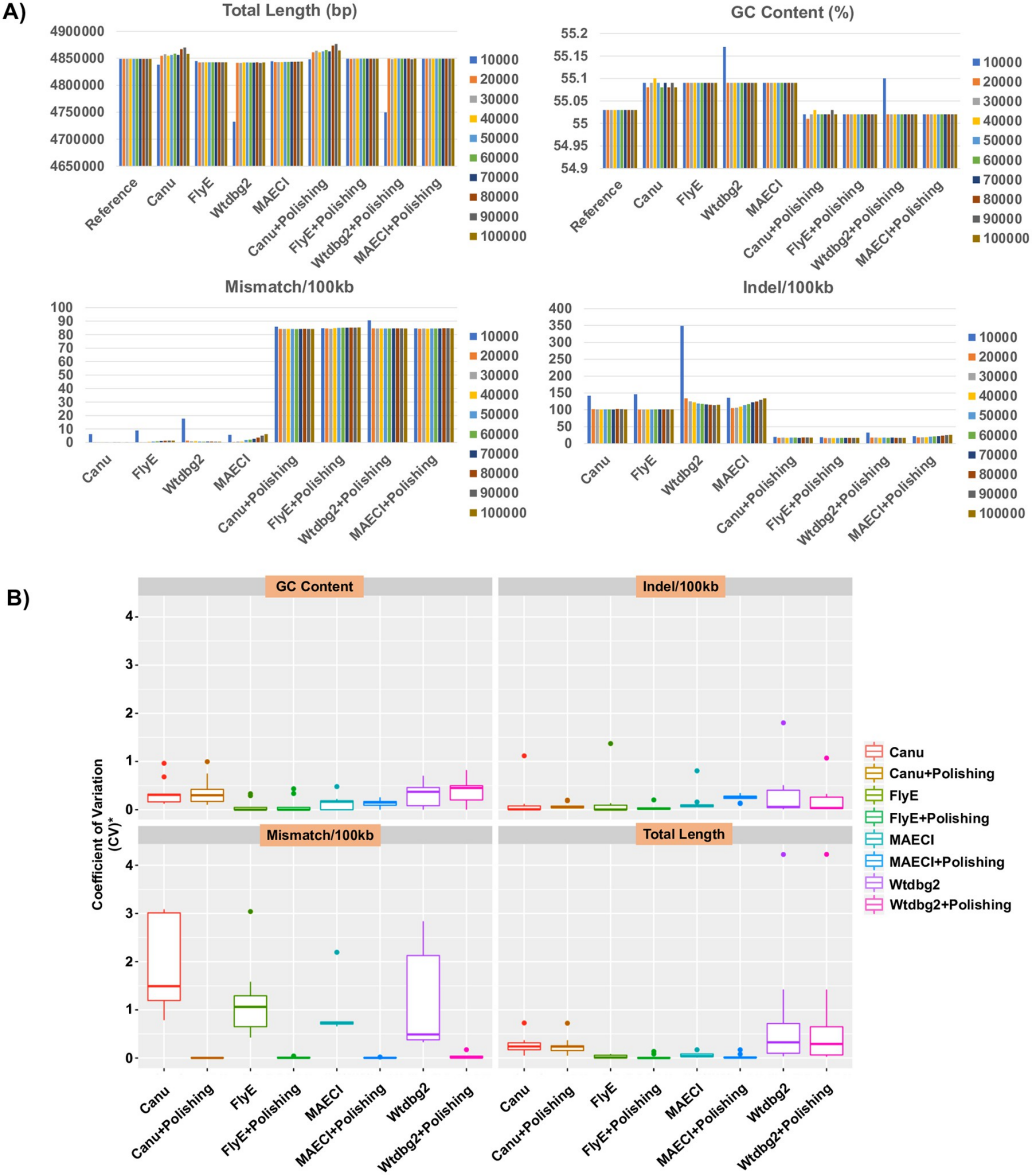
A) Comparison of the performance of Canu, FlyeE, Wtdbg2, and MAECI in the assembly of 10 simulated ONT datasets of *Enterobacter cloacae* strain GGT036 (NZ_CP009756.1) before and after polishing with simulated NGS data. B) Box plots of coefficients of variation (CVs) of total length, GC content, mismatches per 100 kb, and indels per 100 kb from 10 datasets of each of the nine samples. * represents that the smaller CVs have been enlarged: the CVs of the total length were multiplied by 100, and the GC content was multiplied by 1,000.

The contig number, total length, GC content, N50, genome fraction, mismatches per 100 kb, and indels per 100 kb of assemblies from each program were calculated using QUAST (S3 Table in S1 File). The performance of MAECI was basically not affected by data volume, as the results were stable, and the total length and GC content after polishing using NGS data were close to the reference sequences (S1 Fig). For example, the genome of *Enterobacter cloacae* was 4,848,754 bp long with GC content of 55.03% (Fig 2A). Before polishing with NGS data, the coefficients of variation (CVs) of MAECI were 1.18e-04 (mean assembly length: 4,843,237 bp) and 2.719e-16 (mean GC content: 55.09%). After polishing, the CVs were 1.104e-05 (mean assembly length: 4,848,936 bp) and 1.361e-16 (mean GC content: 55.02%), both at the lowest. The CVs of Wtdbg2 were the highest among the four methods. Similarly, the CVs of mismatches per 100 kb of MAECI before and after polishing were 0.69 (mean: 2.945) and 0.0014 (mean: 84.522), respectively, which were the lowest. The indels per 100 kb of MAECI had CV values of 0.088 (mean: 119.668) and 0.12 (mean: 21.349) before and after polishing, respectively. MAECI and FlyE had the lowest CVs of indels per 100 kb before and after polishing, respectively. By comparing and analyzing the statistical results of indels per 100 kb of Wtdbg2 and MAECI before NGS polishing, we found that when the number of reads is less than 40,000, the performance of MAECI is better than that of Wtdbg2, while when the number of reads is more than 40,000, the result is opposite (S1 Fig). This phenomenon may indicate that when using nanopore sequencing technology to assemble bacterial genomes, the read number may also affect the assembly results, not the more the better. After all, the greater the more sequencing reads, the higher the number of sequencing errors. We also analyzed the CVs of 10 datasets from each of the nine samples for total length, GC content, mismatches per 100 kb, and indels per 100 kb. We found that MAECI, with or without polishing with NGS data, showed low variation with different sequencing data volumes (Fig 2B).

### 3.2 Comparison of assembly results from published data

**Fig 3:**
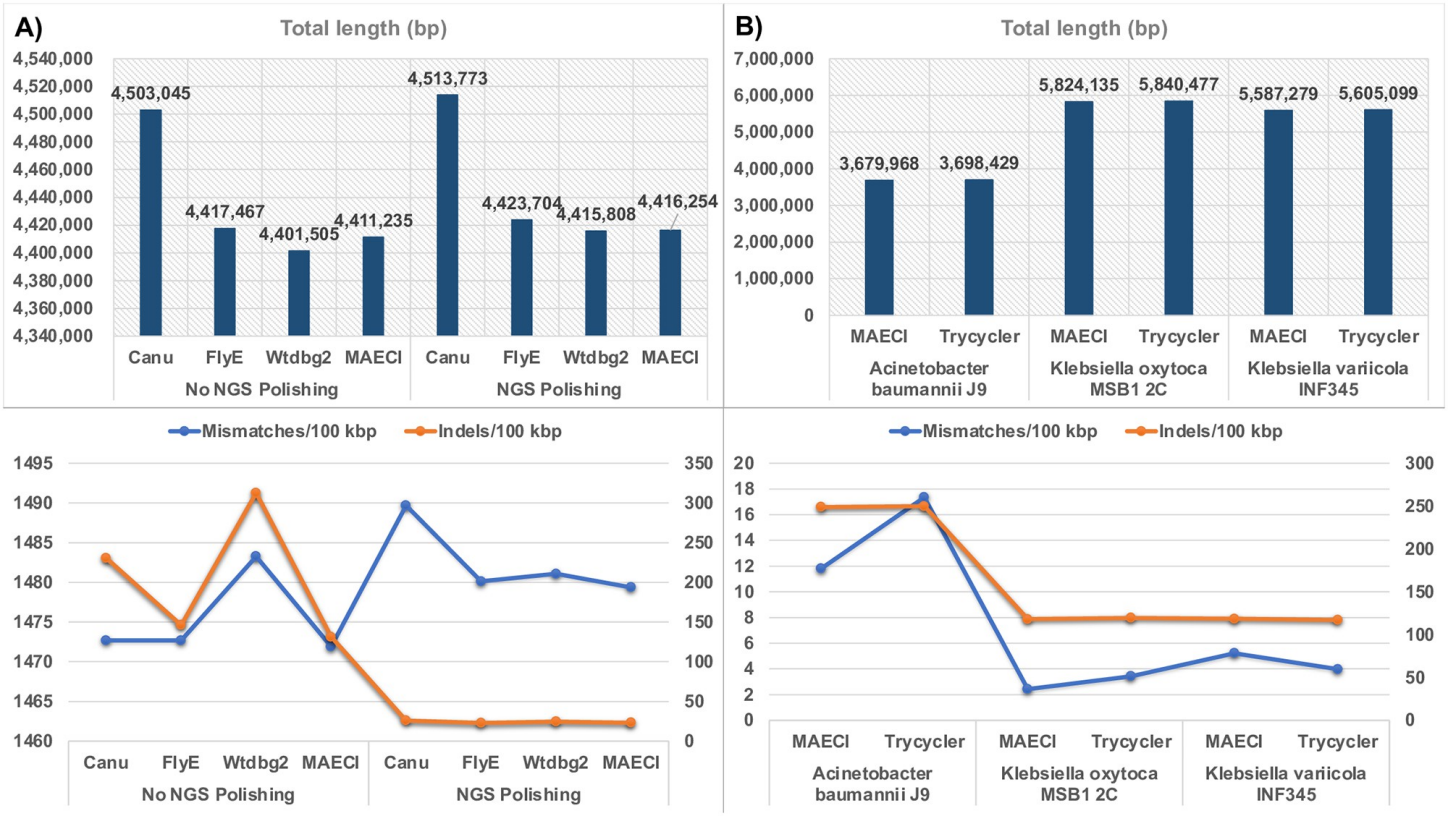
A) Performance comparison of Canu, FlyE, Wtdbg2 and MAECI on real datasets of *A*. *faecalis* PGB1 published by *Lang et al*. B) Comparison of MAECI and Trycycler on real ONT datasets of three strains published by *Wick et al*.

We performed the assembly analysis and comparison of results using the default parameters of MAECI on the *Lang et al*.’s data (Fig 3A, S4 Table in S1 File). The paper disclosed that the genome size of *A*. *faecalis* PGB1 was 4,414,056 bp (4,239,915 bp circular chromosome and 174,141 bp plasmid). Without any manual processing for the assembly results, compared with Cany, FlyE and Wtdbg2, the assembled genome length of MAECI before NGS polishing was the closest to the conclusion of the paper, and the second closest after NGS polishing. No matter whether NGS polishing was performed or not, MAECI’s mismatches per 100 kb were both the least, which were 1471.9 and 1479.37, respectively. For indels per 100 kb, we found that before NGS polishing, MAECI’s indels per 100 kb were the least, which were 131.34; but after NGS polishing, it reached 23.18, which were the second best. The overall assembly performance of MAECI seemed to be satisfactory, meanwhile, it also confirmed the importance and necessity of polishing the assembly results with NGS data.

The performance of MAECI was evaluated using the ONT data of *Acinetobacter baumannii* J9, *Klebsiella oxytoca* MSB1 2C, and *Klebsiella variicola* INF345 published by *Wick et al*. The number of simulated reads were close to the reported number after filtration using Filtlong (Table 1). We also performed a comparative analysis using the assemblies from Trycycler published in the paper (S5 Table in S1 File). We found the assembly performance of MAECI and Trycycler was essentially the same. MAECI had a lower value of mismatches per 100 kb and indels per 100 kb than Trycycler in *Acinetobacter baumannii* J9, *Klebsiella oxytoca* MSB1 2C, but not in *Klebsiella variicola* INF345 (Fig 3B, S2 Fig), suggesting an improvement in assembly accuracy.

**Table 1:** The simulated data statistics of three bacterial strains.

Sample	Paper_data-Rapid (after Filtlong)			Simulated data		
	Read count	Total size	N50 length	Read count	Total size	N50 length
*Acinetobacter baumannii* J9	69,135	741,274,205	15,130	70,000	543,179,080	9,385
*Klebsiella oxytoca* MSB1 2C	69,978	768,514,620	15,181	70,000	543,767,980	9,411
*Klebsiella variicola* INF345	85,440	948,228,758	15,364	90,000	698,317,203	9,363

## 4. Discussion

MAECI, which combined multiple input assemblies into a consensus sequence and performed self-error correction, produced a more accurate long-read-only assembly sequence. Although the results of MAECI showed the advantages of stability and high efficiency, there are still some shortcomings in the current version. As we known, different assembly software/algorithms have their own advantages and disadvantages, which may have different effects on the assembly results. For example, Canu was reliable and did well with plasmids, but may be the slowest assembler and suffered from large circularization problems. FlyE was a strong and well-balanced performer, which was reliable, robust, good with plasmids and the fewest large-scale indel errors, but it often deleted some sequence when circularizing contigs and had the higher RAM usage. Wtdbg2 ran very fast and saved a lot of computational resources, but it may also miss some sequence which could cause incomplete assembly results. Raven suffered from worse circularization problems than FlyE and was not good with small plasmids, although which was reliable, robust for chromosome assembly and cost very little RAM [8]. Therefore, in the present study, only three assembly software (Canu, FlyE and Wtdbg2) were employed, and other software and/or algorithm such as Raven and La Jolla Assembler (LJA) [25] can be included for analysis, optimization and performance comparison in the next work. However, is it true that the more assembly software/algorithms and error correction methods are included, or the better software combination under the same conditions is selected, the better the performance of MAECI will be and the higher the accuracy of the assembly results will be? This may be something we need to carefully consider and focus on in the next work, which may require extensive software/algorithms and/or software combination test analysis to evaluate. After polishing with NGS data, mismatches per 100 kb increased, but indels per 100 kb decreased significantly. Although this may be explained by the fact that the error rate and base or read quality score of simulated data were both “K”, the specific reasons are not further discussed in this study but will be a focus of our future research. Due to the principle of MAECI, the assembly results of MAECI may be easily affected by the selected assembly software with the least number of scaffold result, the integrity of the assembly results of such software will be very critical and needs to be carefully evaluated, although the problem may not be particularly apparent for long-read data by nanopore sequencing technology. The design principle of MAECI may be similar to that of Trycycler, but there are significant differences. For example, Trycycler is not an automated pipeline and recommends users generate 12 independent input assemblies, while MAECI is a more automated analysis pipeline and can directly input nanopore sequencing data for analysis. The input assemblies by Trycycler needs to be complete, that is, one contig per replicon, and uses MUSCLE to produce a multiple sequence alignment and the results may depend on the result of the multiple sequence alignment, while MAECI is not required and uses Racon to generate consensus sequence. For the software running time, the software included in the current version of MAECI is running one by one, which is bound to cause time consumption. This also means that MAECI may need more computational resources and time than single-assembler assemblies, so there could be some requirements for computing hardware. We will also optimize for this problem, such as parallel analysis of the assembly process, thereby reducing the overall assembly run time.

Overall, we hope that MAECI can help to improve the accuracy of bacterial genome assembly and provide a reference solution for bacterial genome assembly. With continuous optimization for MAECI, improvements in nanopore sequencing technology, basecalling model, assembly algorithms and/or polishing/error correction algorithms, it may be possible to make perfect bacterial assembly a reality.

## 5. Conclusions

MAECI is an efficient and effective pipeline for the assembly of bacterial genomes. It can be used in the assembly and analysis of bacterial and small genomes as an integration pipeline and supplementary method to genome assembly methods. With further development and optimization, we hope that this pipeline can be used in a wider range of scenarios and even in the assembly of eukaryotic or polyploid genomes.

## Supporting information

S1 Fig Statistical analysis of 9 simulated data assembly results. (PDF)

S2 Fig Comparison of MAECI and Trycycler on real ONT datasets of three strains published by *Wick et al*. (PDF)

S1 File Supplementary sheet. (XLSX)
