# Supplementary material for: MAECI: A pipeline for generating consensus sequence with nanopore sequencing long-read assembly and error correction
Source: PLoS One. 2022 May 20;17(5):e0267066. doi: 10.1371/journal.pone.0267066 (PMC9122195; doi:10.1371/journal.pone.0267066)

Figure S1. Statistical analysis of 9 simulated data assembly results.

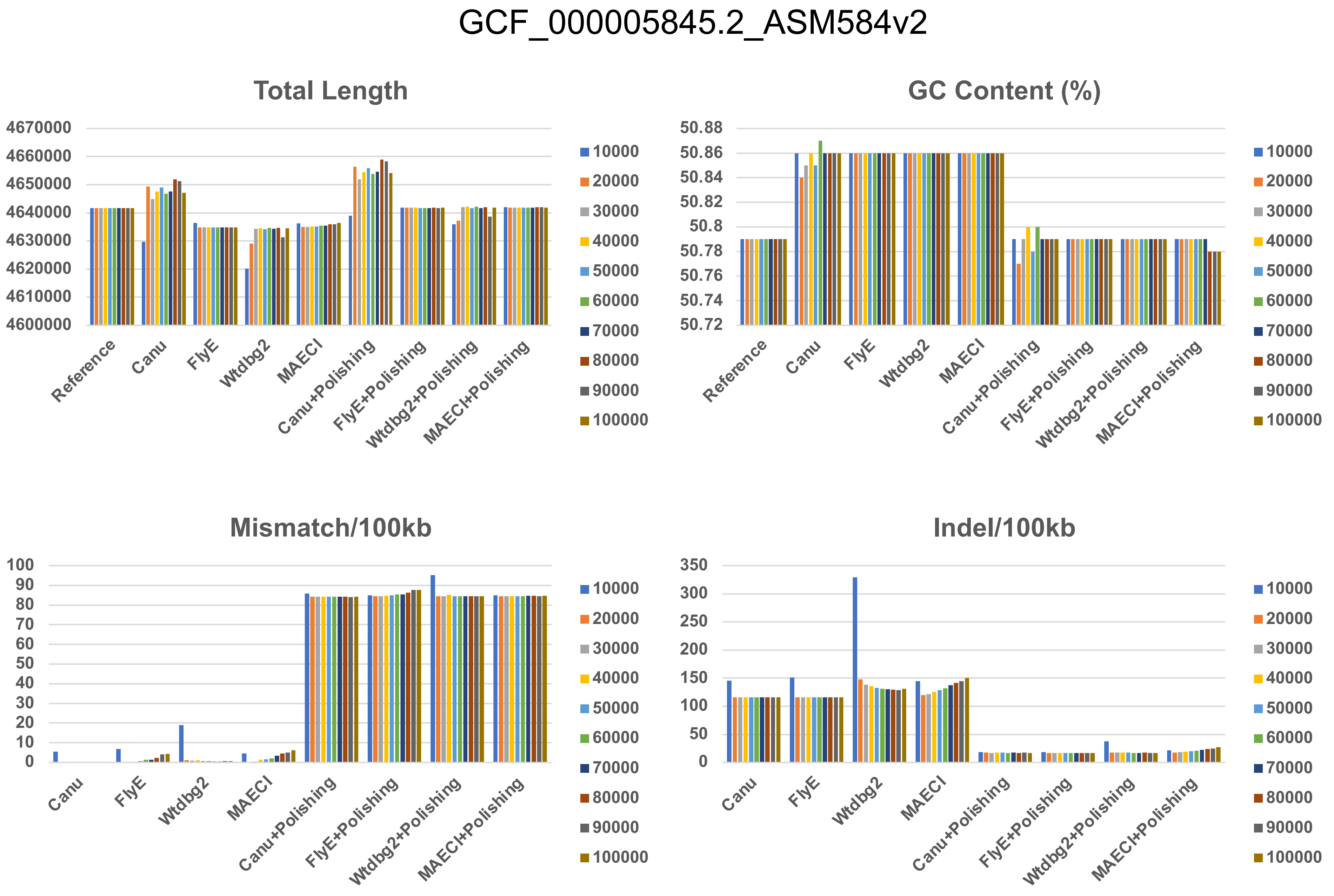

GCF_000006765.1_ASM676v1

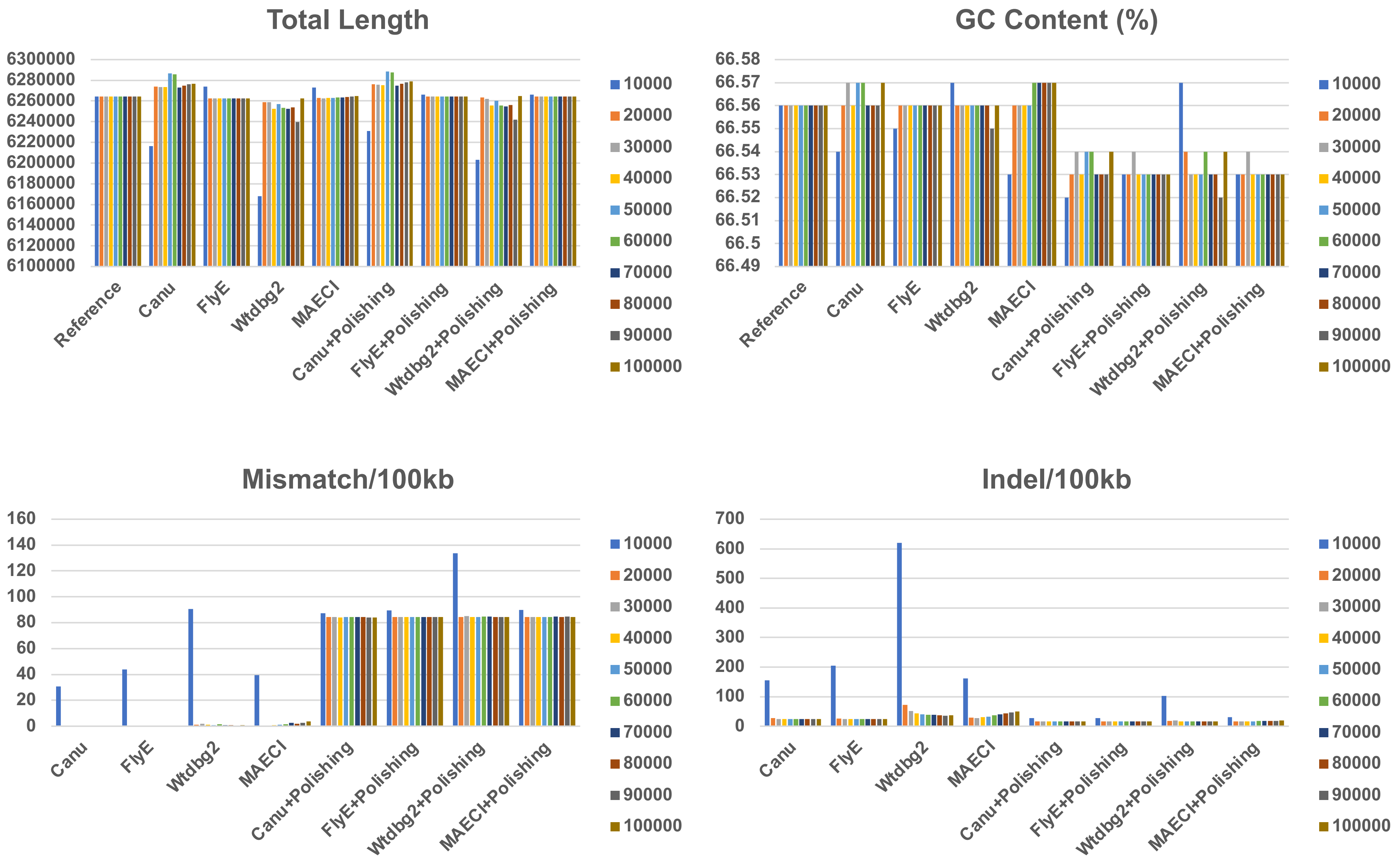

GCF_000009085.1_ASM908v1

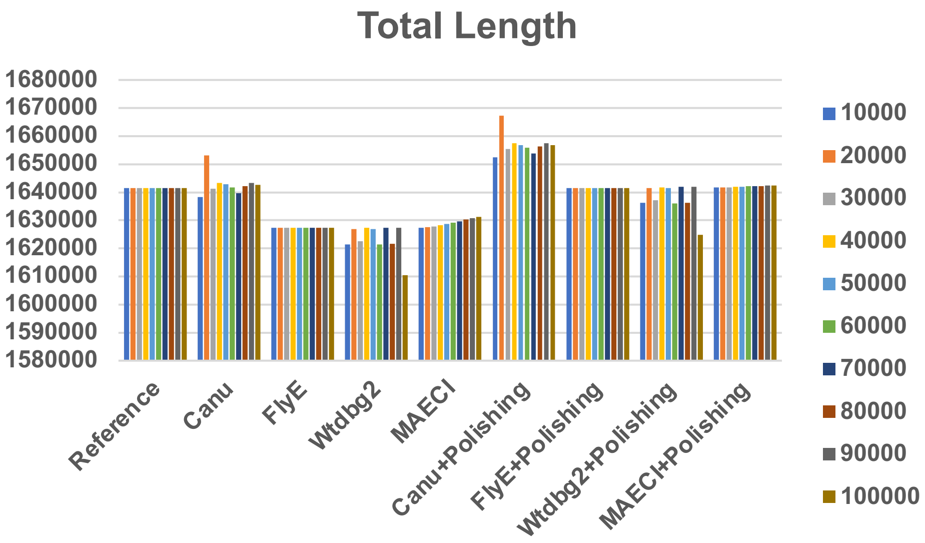

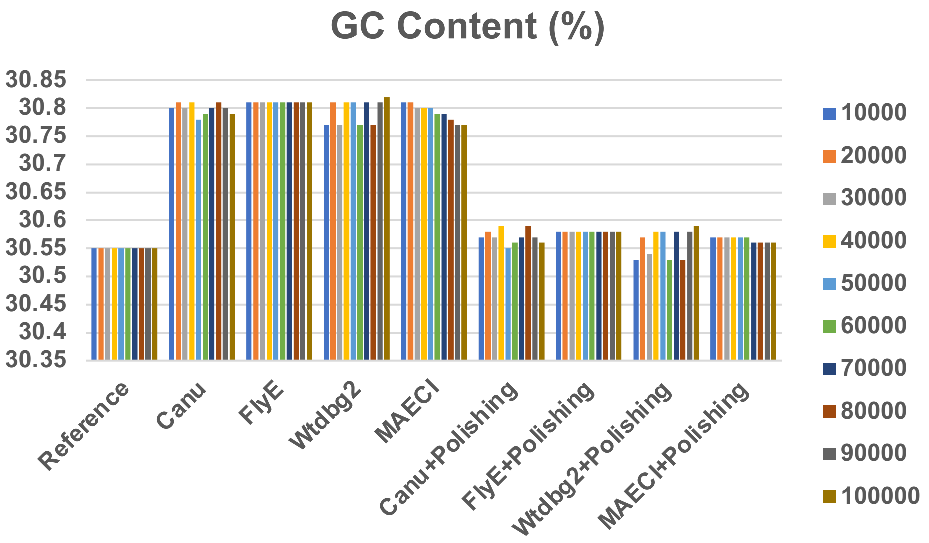

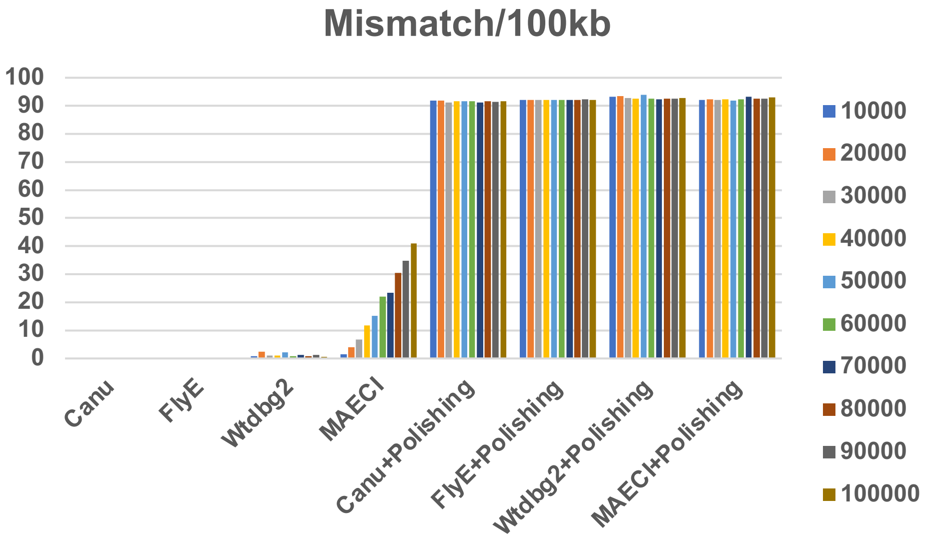

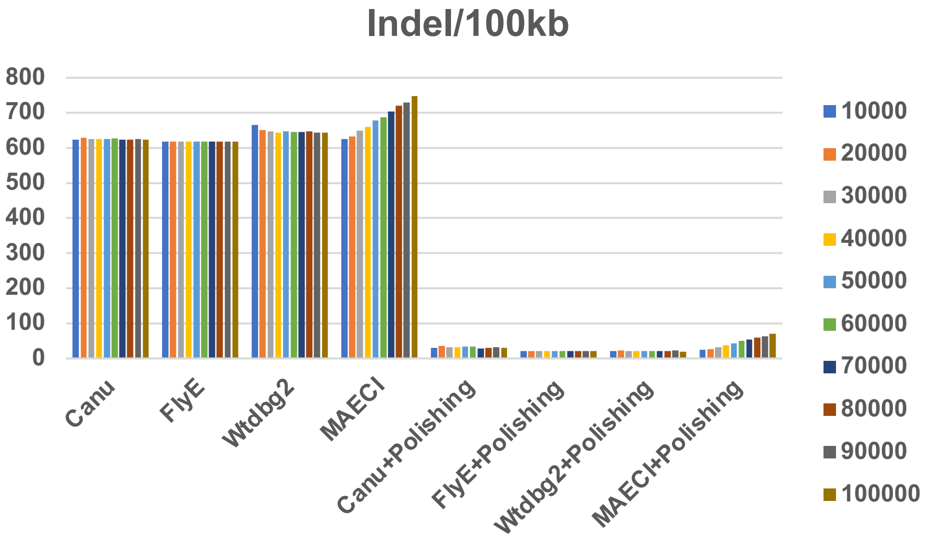

GCF_000013285.1_ASM1328v1

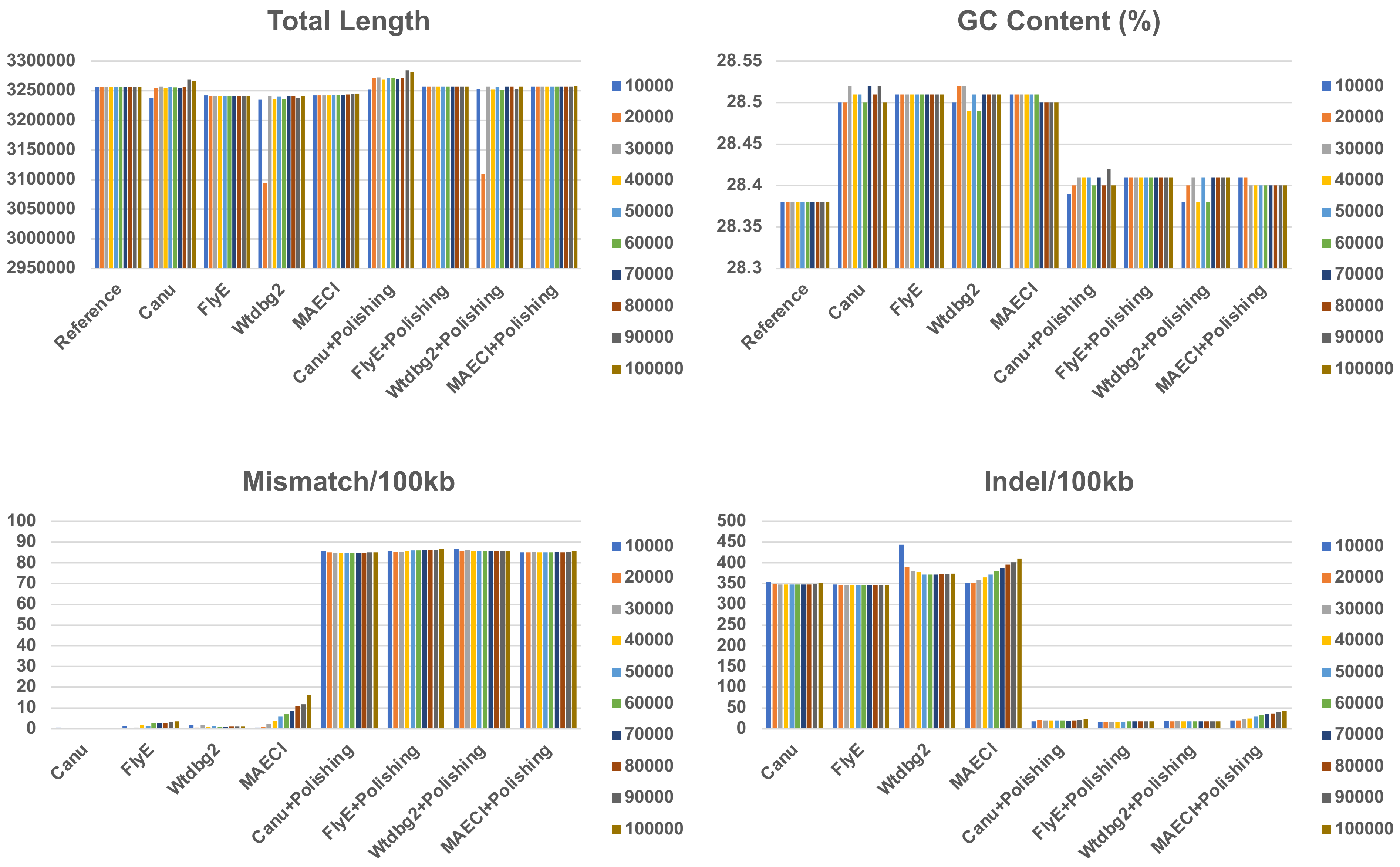

GCF_000013425.1_ASM1342v1

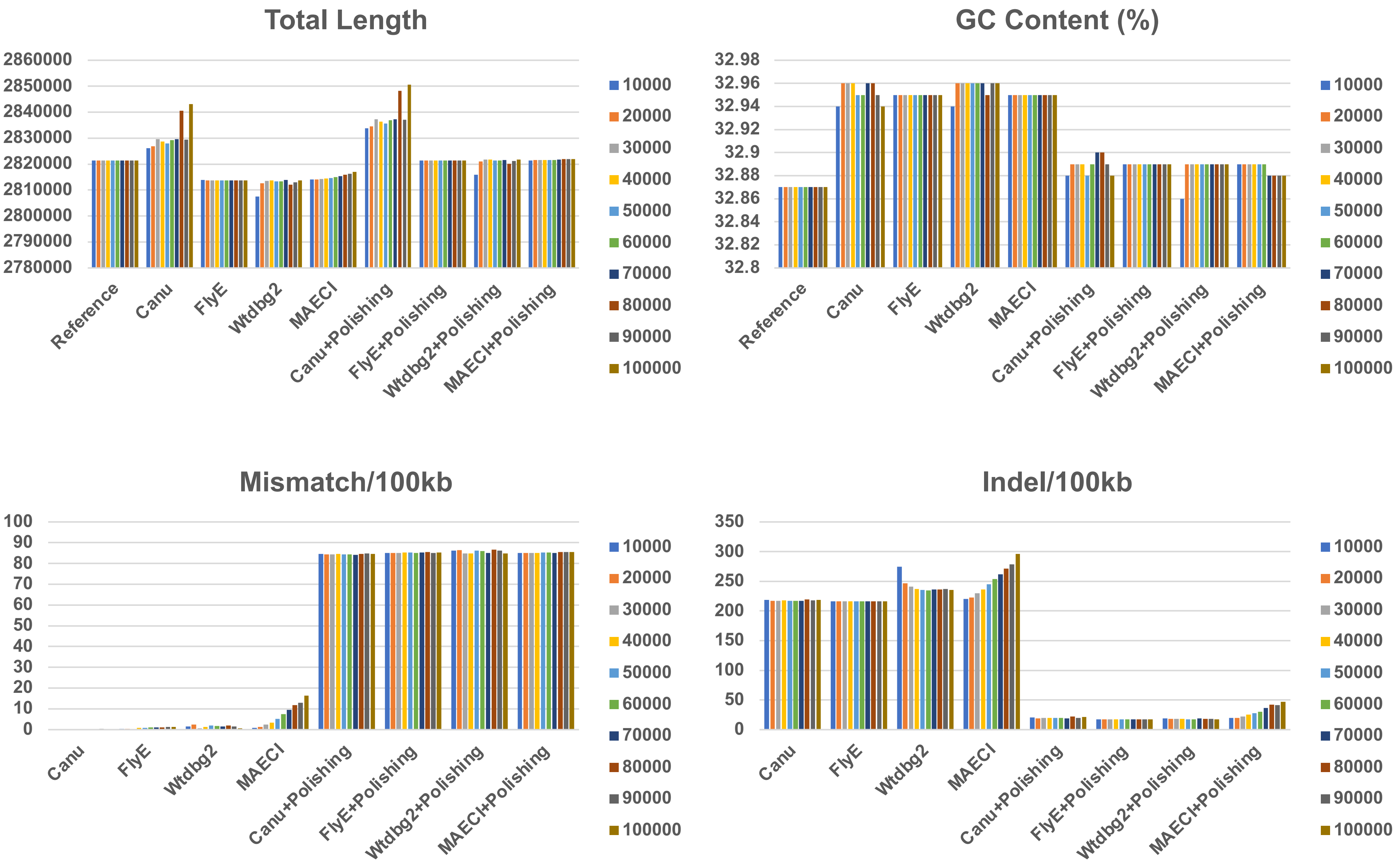

GCF_000196035.1_ASM19603v1

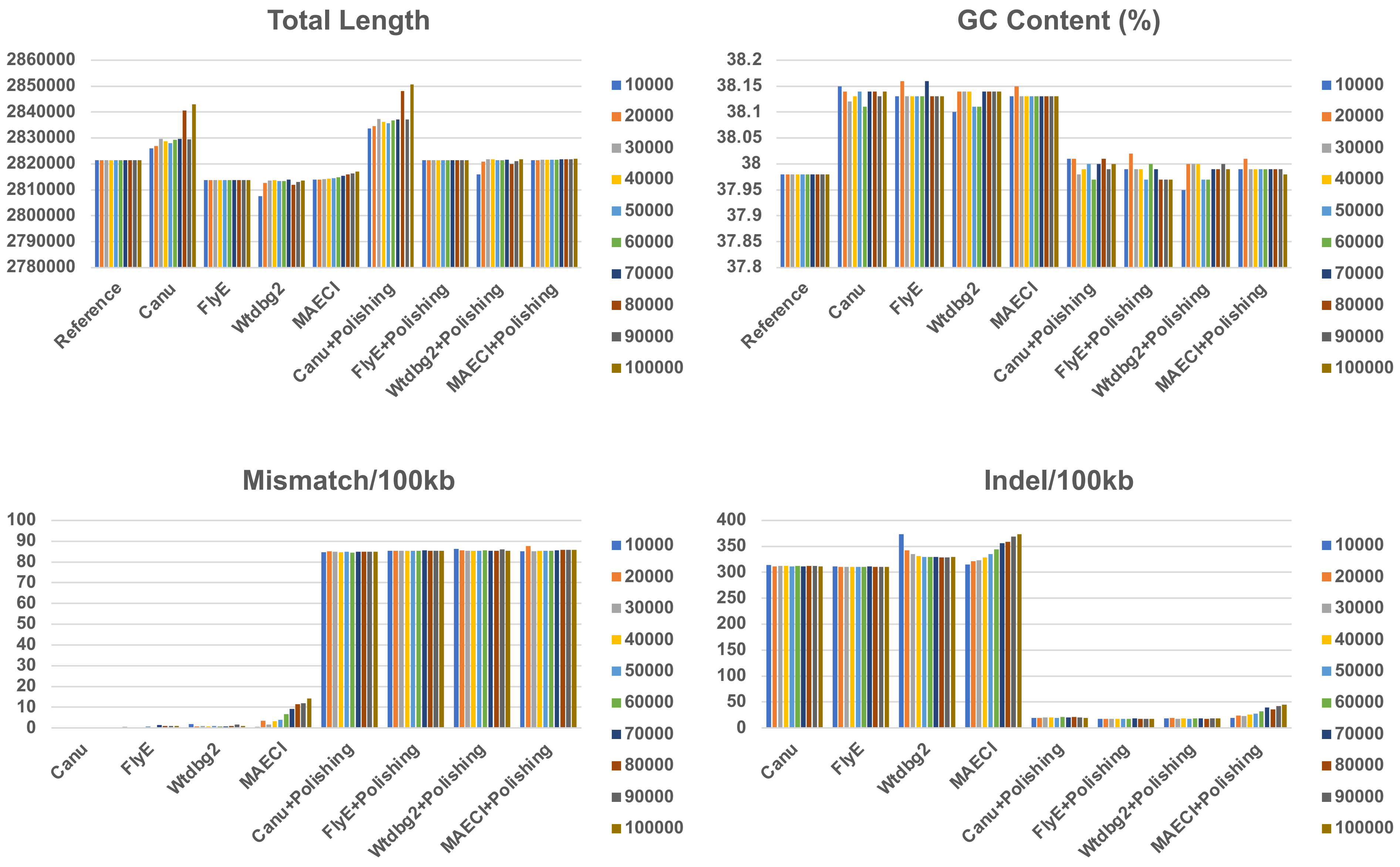

GCF_000770155.1_ASM77015v1

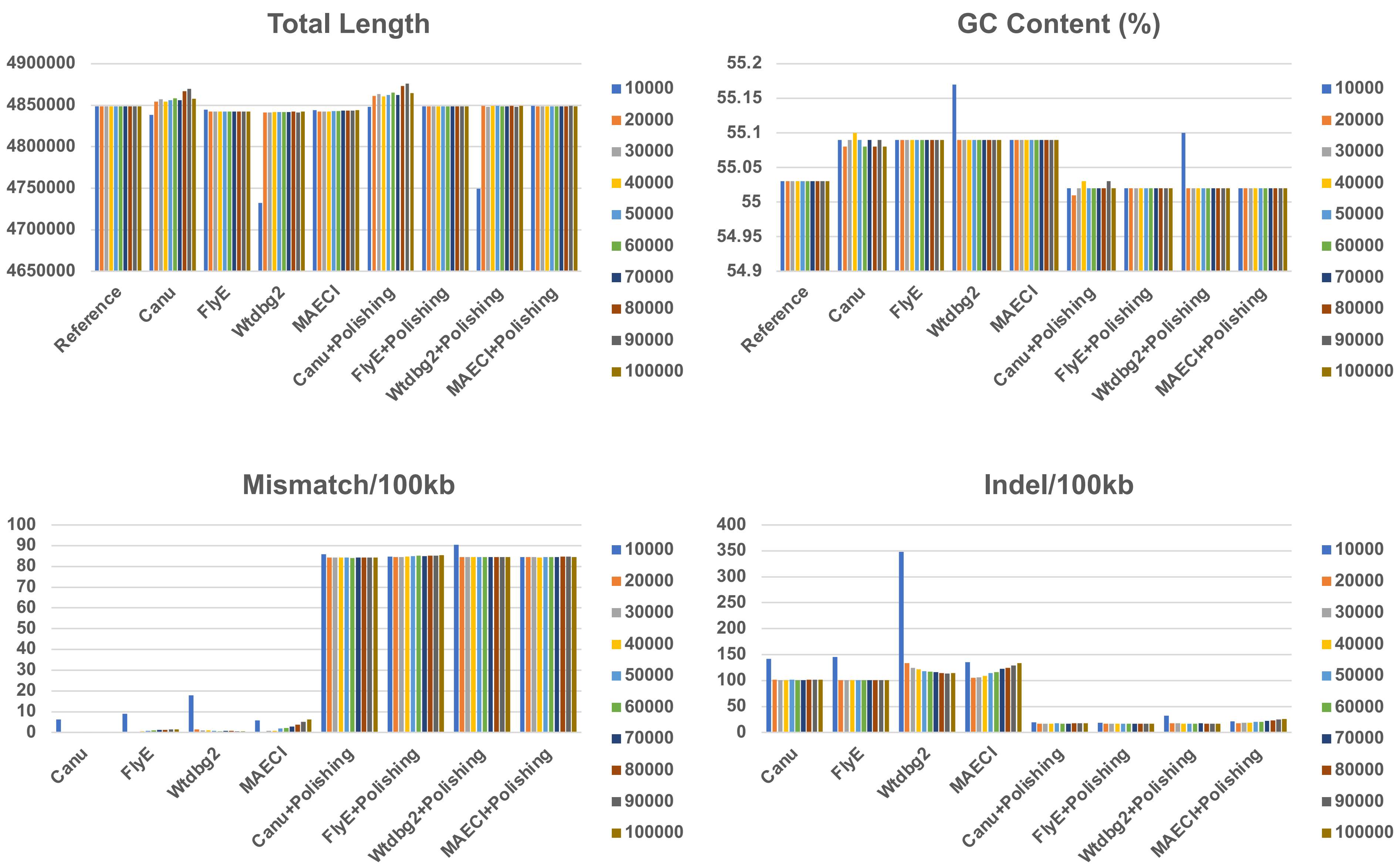

GCF_003312465.1_ASM331246v1

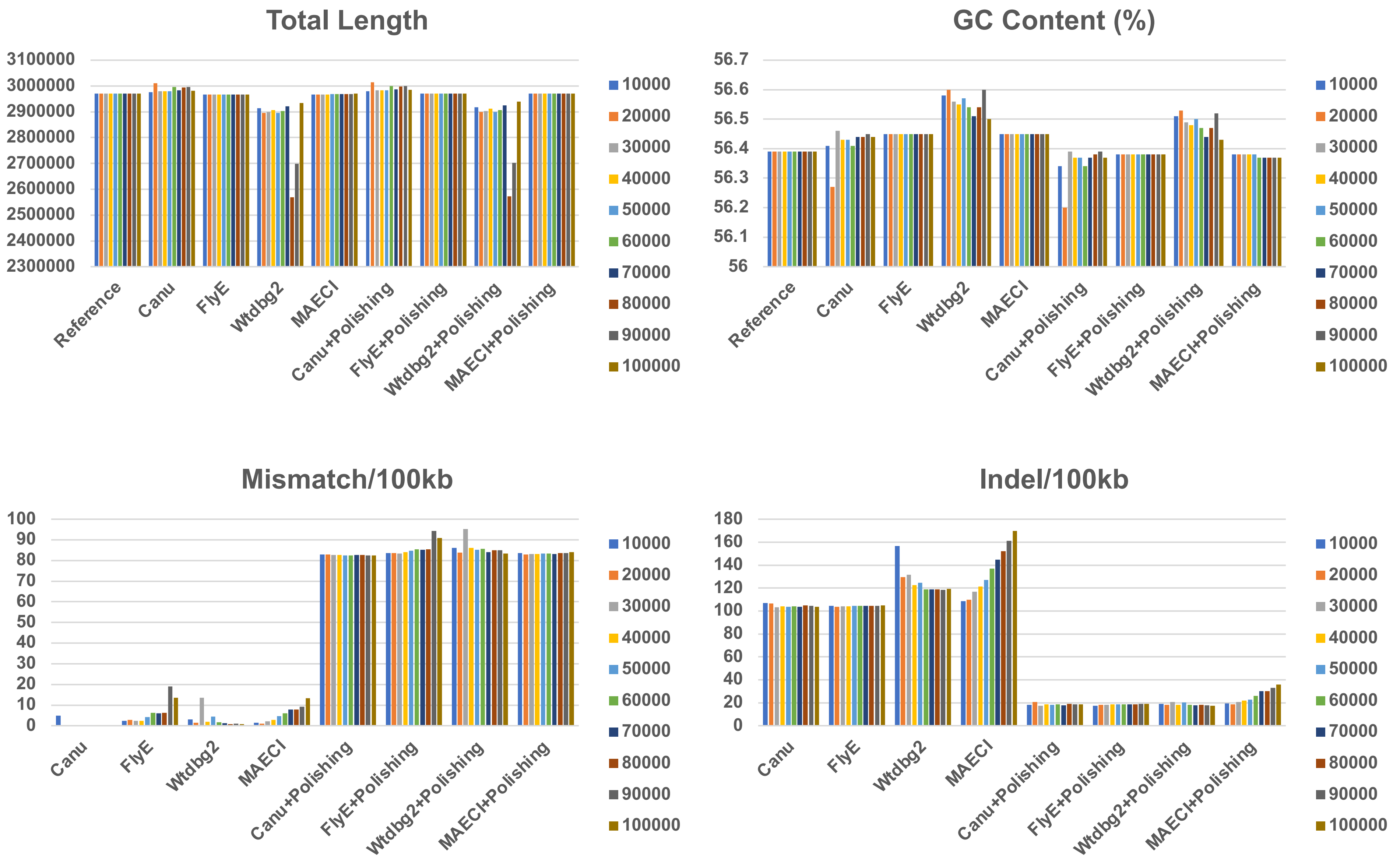

GCF_003319815.1_ASM331981v1

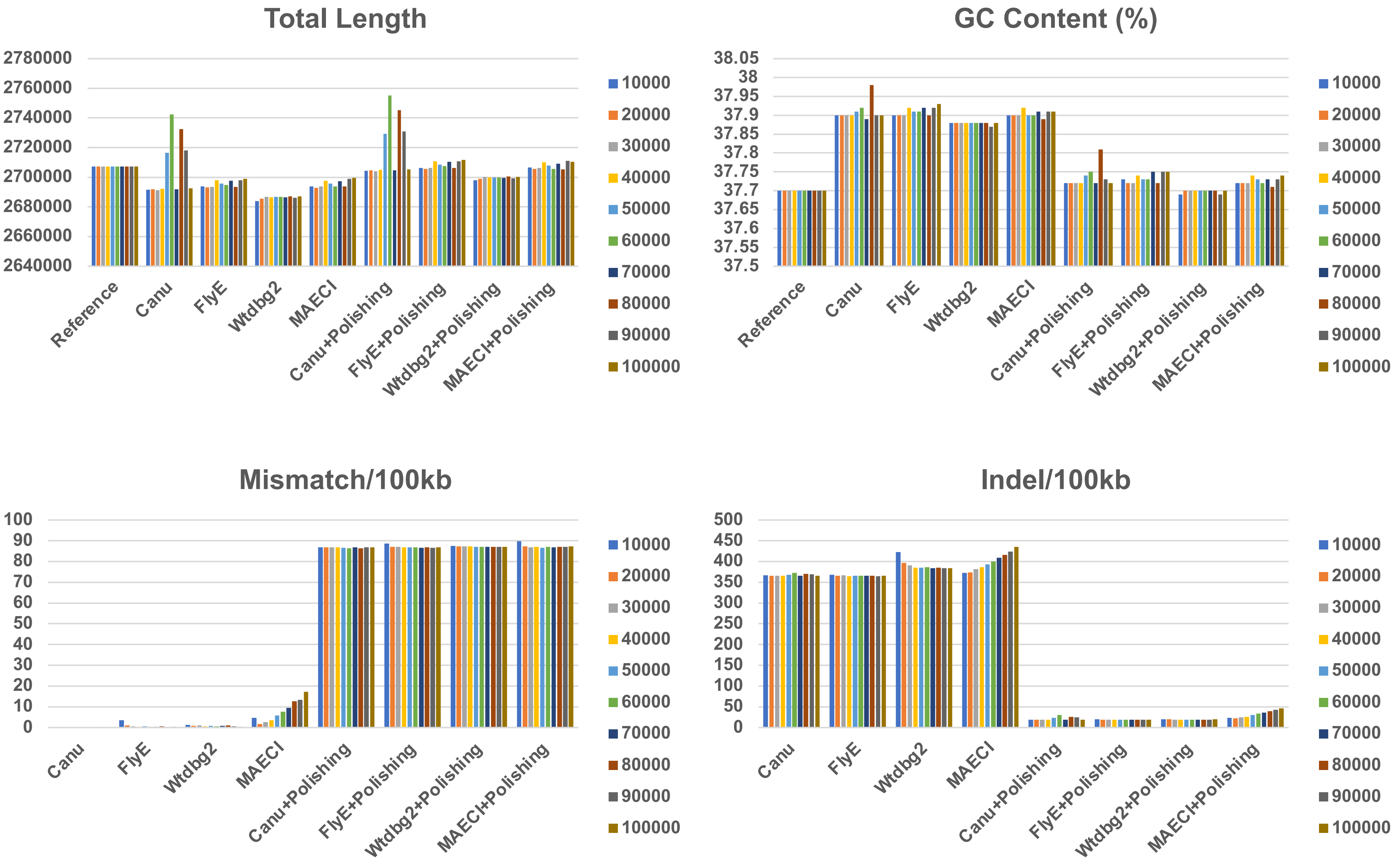

Supplement: S1 Fig — (PDF) [file pone.0267066.s001.pdf]
