# Supplementary material for: MAECI: A pipeline for generating consensus sequence with nanopore sequencing long-read assembly and error correction
Source: PLoS One. 2022 May 20;17(5):e0267066. doi: 10.1371/journal.pone.0267066 (PMC9122195; doi:10.1371/journal.pone.0267066)

Figure S2. Comparison of MAECI and Trycycler on real ONT datasets of three strains published by *Wick et al*.

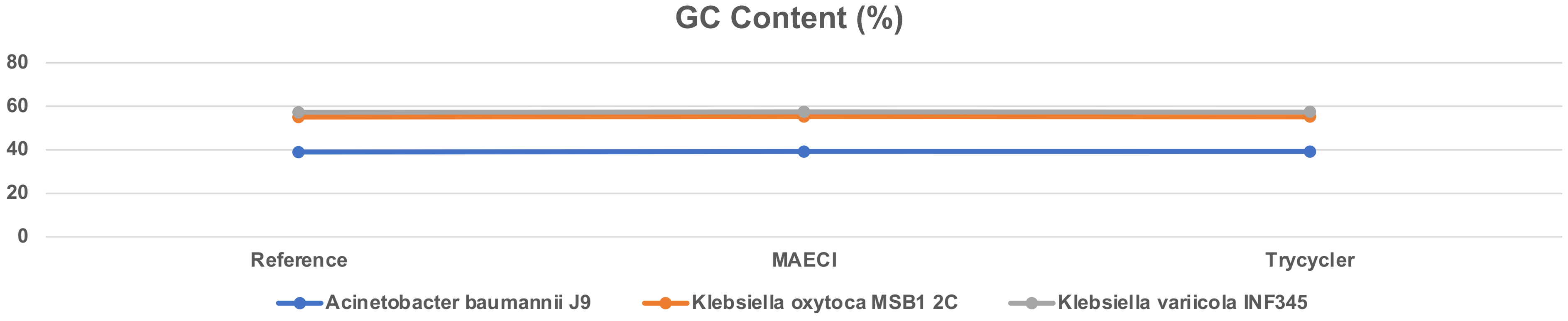

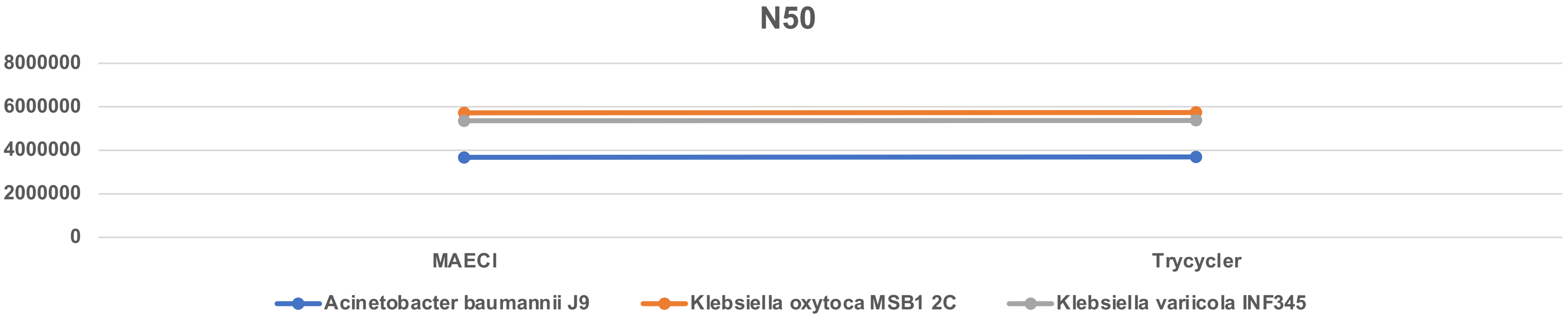

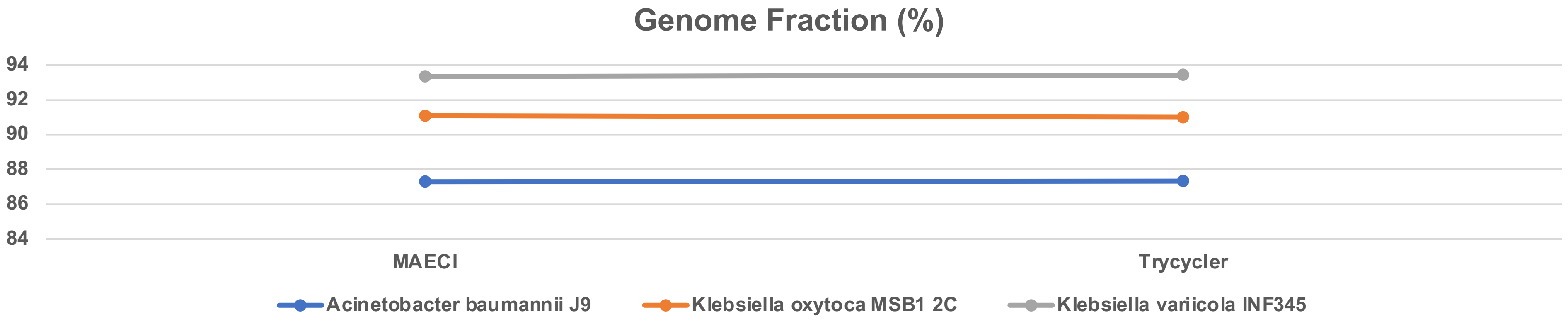

Supplement: S2 Fig — (PDF) [file pone.0267066.s002.pdf]
